# Effects of Buffer Size and Shape on the Association of Neighborhood SES and Adult Fruit and Vegetable Consumption

**DOI:** 10.3389/fpubh.2021.706151

**Published:** 2021-11-10

**Authors:** Minal Patel, April Y. Oh, Laura A. Dwyer, Heather D'Angelo, David G. Stinchcomb, Benmei Liu, Mandi Yu, Linda C. Nebeling

**Affiliations:** ^1^Behavioral Research Program, Division of Cancer Control and Population Sciences, National Cancer Institute, National Institutes of Health, Bethesda, MD, United States; ^2^Cape Fox Facilities Services, Manassas, VA, United States; ^3^Westat, Inc., Rockville, MD, United States; ^4^Surveillance Research Program, Division of Cancer Control and Population Sciences, National Cancer Institute, National Institutes of Health, Bethesda, MD, United States

**Keywords:** socioeconomic factors, environment, fruit and vegetables, diet, geospatial research

## Abstract

**Introduction:** Neighborhood environment factors are relevant for dietary behaviors, but associations between home neighborhood context and disease prevention behaviors vary depending on the definition of neighborhood. The present study uses a publicly available dataset to examine whether associations between neighborhood socioeconomic status (NSES) and fruit/vegetable (FV) consumption vary when NSES is defined by different neighborhood sizes and shapes.

**Methods:** We analyzed data from 1,736 adults with data in GeoFLASHE, a geospatial extension of the National Cancer Institute's Family Life, Activity, Sun, Health, and Eating Study (FLASHE). We examined correlations of NSES values across neighborhood buffer shapes (circular or street network) and sizes (ranging from 400 to 1,200 m) and ran weighted simple and multivariable regressions modeling frequency of FV consumption by NSES for each neighborhood definition. Regressions were also stratified by gender.

**Results:** NSES measures were highly correlated across various neighborhood buffer definitions. In models adjusted for socio-demographics, circular buffers of all sizes and street buffers 750 m and larger were significantly associated with FV consumption frequency for women only.

**Conclusion:** NSES may be particularly relevant for women's FV consumption, and further research can examine whether these associations are explained by access to food stores, food shopping behavior, and/or psychosocial variables. Although different NSES buffers are highly correlated, researchers should conceptually determine spatial areas a priori.

## Introduction

Healthy eating patterns predict a lower risk of obesity, cardiovascular disease, cancer, and diabetes (1). However, fruit and vegetable (FV) consumption among adults in the United States falls below recommended guidelines (1). Neighborhood socioeconomic status (NSES) is one factor that may be relevant for understanding dietary behaviors and obesity. For example, lower, vs. higher, income neighborhoods have fewer supermarkets, more convenience stores, and fewer healthy food options (2, 3), and higher NSES has been associated with increased consumption of fruits and vegetables (4). However, some studies have found inconsistent associations.

Differences regarding the impact of NSES on energy balance could be attributed to differences in defining the size and shape of a neighborhood (5). This issue (6) has been observed in analyses of NSES and the food environment (7) and is important to consider for understanding whether a true relationship between the neighborhood-level factor and behavioral outcome exists, or whether it is an artifact of how the neighborhood-level factor is defined.

Neighborhoods are often defined using existing administrative boundaries such as census tracts, although census tract boundaries may not reflect one's lived experience (5). Other methods include tracking individual transportation behavior or using community based approaches to have individuals draw neighborhood boundaries on a map (8). Another approach involves generating buffers that define the space of the neighborhood as a specific distance from a location (e.g., a specific distance from a person's home) in all directions. This approach to defining neighborhood based on distance from the participant's home was used in the present study.

There is not consensus on the most appropriate buffers for studies of NSES and FV consumption (9–11). For example, it is unclear from existing literature what matters more for FV consumption: buffers defined as circular in shape (home neighborhood conceptualized as a radial distance from the home in all directions), vs. buffers defined along the street network (home neighborhood conceptualized as a distance from the home in all directions that reflects the distance that one would travel if following the local streets). Related, there is not consensus about whether home neighborhood matters more for FV consumption when defined as a small neighborhood (e.g., 400 m from the home) vs. when a larger neighborhood is considered (e.g., 1,200 m from the home).

Therefore, our objective was to examine the neighborhood size(s) and shape(s) that are important for understanding the relationship of NSES and FV consumption. We anticipated fewer significant associations between NSES and FV consumption frequency as the definition of neighborhood area size increases, because smaller neighborhoods may be more relevant to everyday lived experiences. We also explored whether these associations varied by gender. Associations between neighborhood environment variables and health-related domains may differ for women and men. For example, one US-based study found that individual education level was more strongly associated with FV consumption among women (but not men) who lived in lower poverty neighborhoods as compared to higher poverty neighborhoods (12). Additional research has shown that effects of neighborhood variables (e.g., socioeconomic status, cohesion, and resources) on other health-related domains (for example, physical activity, self-rated health) can vary by gender (13, 14). Combined with research demonstrating gender differences in amount of FV consumption and related behaviors such as food shopping (15, 16), we examined whether NSES-FV associations at all neighborhood definitions differed across gender.

## Methods

We used data from the Family Life, Activity, Sun, Health, and Eating Study (FLASHE) study, a cross-sectional study sponsored by the National Cancer Institute on correlates of cancer-preventive behaviors among dyads of parents/caregivers and their adolescent children residing in the United States (17). FLASHE self-report data were collected in April–October 2014 *via* two web-based surveys focused on (a) diet-related behaviors, and (b) physical activity-related behaviors. Surveys were administered to both parents and adolescents (17, 18). The Institutional Review Boards of Westat, Inc., and the National Cancer Institute approved the FLASHE study protocol.

**Figure 1:**
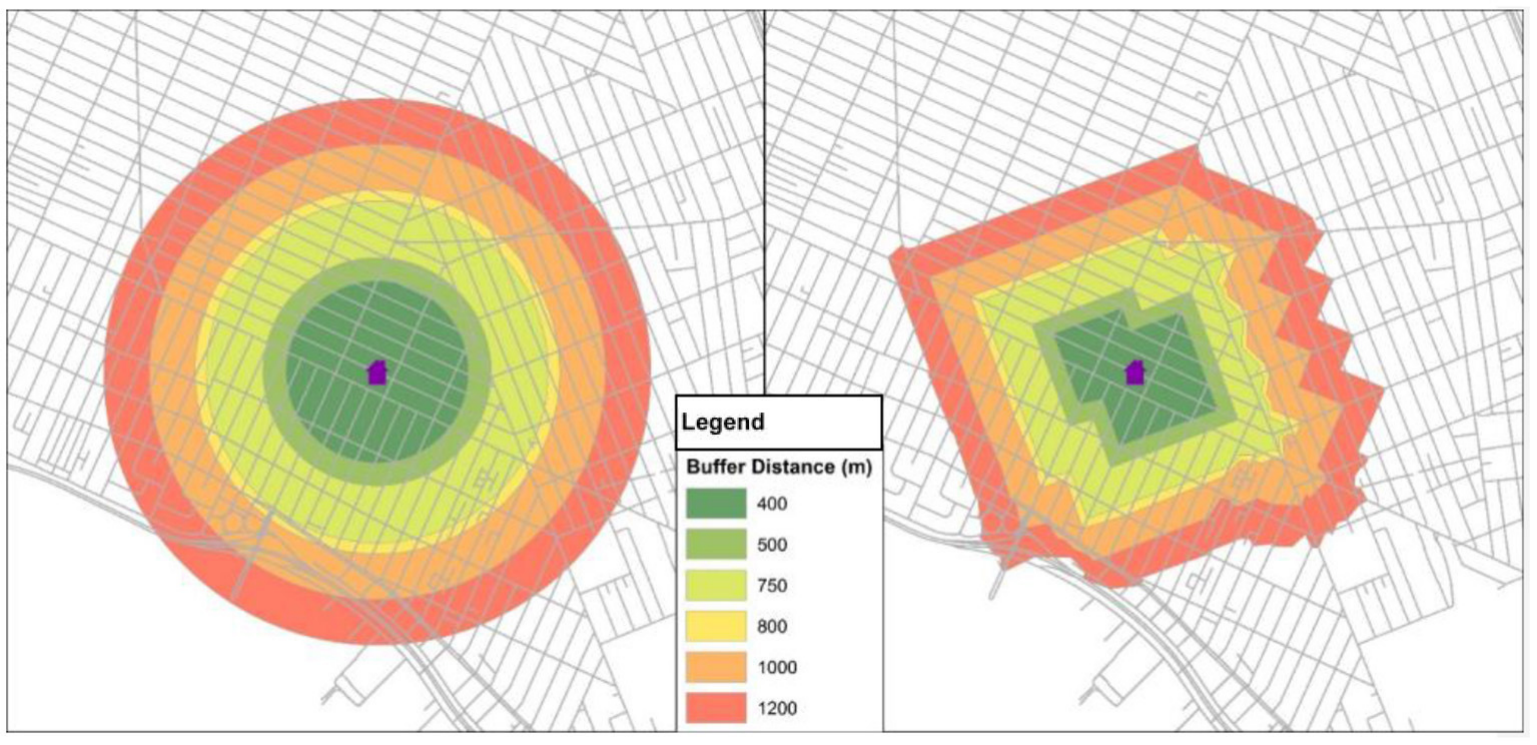
Neighborhood definitions in the GeoFLASHE dataset: Example of Circular and Street Network Buffers. The example circular buffers **(Left)** and street network buffers **(Right)** are shown for various distances from the participants' home location ranging from 400 to 1,200 m. The publicly available GeoFLASHE dataset includes neighborhood variables for each of these 12 neighborhood definitions varying in buffer shape (circular, street network) and size (400, 500, 750, 800, 1,000, and 1,200 m from the participant's home location) (20).

The publicly available FLASHE resources include GeoFLASHE, a set of calculated neighborhood variables that can be merged with survey data. To calculate these variables, residential addresses for each dyad were geocoded. Neighborhood was defined in the FLASHE surveys as a 10–15 min walk from an individual's home, and the calculations for GeoFLASHE were based on an assumption of this distance at an average pace of 20 min/mile (19). A range of neighborhood buffers were calculated around the dyads' home at six different sizes (400, 500, 750, 800, 1,000, and 1,200 m). We calculated two sets of neighborhood buffers: circular buffers (radial distances drawn from the home address, not accounting for street networks or other natural barriers) and street network buffers (incorporating intersection and road network data, and generated using the TIGER 2010 road network in ArcGIS [Streetmap USA, ESRI]), as shown in Figure 1. Network buffers tend to be smaller than circular buffers of the same distance due to the structure of street networks. To compute census-based measures at every buffer size and shape, a buffer percentage was first calculated to determine the percentage of each buffer area (e.g., 400 m circular buffer; 1,200 m network buffer) covered by each census tract that intersected the buffer. These buffer percentages were then used to calculate weighted averages of each neighborhood variable in GeoFLASHE (20).

### Participants

FLASHE participants were sampled from the Ipsos Consumer Opinion Panel. Although the sample was not nationally representative, efforts were made to balance the sample for similarity to the U.S. population on demographic characteristics, and survey weights were developed for use in weighted analyses with the goal of approximating similarity to the general US population (18). A total of 1,945 dyads of parents and adolescents (ages 12–17) enrolled in FLASHE, and neighborhood variables for 1,736 dyads, are available in the GeoFLASHE dataset. Inclusion criteria included having complete data for all variables in the analysis, and the present analyses analyzed data from 1,600 adult participants.

### Measures

#### Neighborhood Socioeconomic Status

Data from the US American Community Survey were used to calculate a composite measure of seven census indicators representing six domains known as the Yost SES Index, with a higher score representing greater NSES (21). The domains used in generating the NSES index were measured as follows: (1) Occupation (% working class), (2) Unemployment status (% aged ≥ 16 years who are unemployed), (3) Poverty (% of persons below 150% of poverty line), (4) Income (median household income), (5) Education (average years of schooling estimated as the % of persons at specific education levels, weighted by the school years it takes to achieve their education [18]), and (6) Housing (median house value and median rent). A continuous NSES index score was computed for each buffer size and shape, and it was categorized into equal population quintiles to describe the sample. A continuous NSES score was also calculated at the census tract level for comparison purposes.

#### Frequency of FV Consumption

Items administered in FLASHE surveys were modified from the Dietary Screener Questionnaire used in the 2009–2010 National Health and Nutrition Examination Survey (NHANES) (22) and National Youth Physical Activity and Nutrition Survey. Three items hypothesized to be most relevant to the neighborhood environmental context were analyzed in the present analysis as a proxy measure of FV consumption frequency: “During the past 7 days, how many times did you eat… (1) FRUIT like apples, bananas, melon, etc.? COUNT fresh, frozen, canned, and dried fruit. DON'T COUNT fruit juices… (2) a GREEN SALAD, with or without other vegetables?. (3) other NON-FRIED VEGETABLES like carrots, broccoli, collards, green beans, corn, etc.? DON'T COUNT green salad or potatoes.” Response options were: I did not eat [item] during the past 7 days; 1–3 times in the past 7 days; 4–6 times in the past 7 days; 1 time per day; 2 times per day; and 3 or more times per day. Categories were recoded into daily frequency, using the middle value of response options that included a frequency range [for example, 1–3 times during the past 7 days became a daily frequency of 0.29 after taking the middle value (2) and dividing by 7 days per week]. The daily frequencies of the three fruit/vegetable items were then summed (23).

#### Socio-Demographics

Covariates included: age (18–34; 35–44; 45–59; and ages 60+), gender (gender was assessed as “Are you male or female”), race/ethnicity (Hispanic; Non-Hispanic White; Non-Hispanic Black; and Other), education (high school degree, GED or less; some college but not a college degree; and a 4-year college degree or higher) and urbanicity. Urbanicity was categorized into three categories: urban, suburban, and rural, based on Census 2010 urban and rural area determinations using the individual's home address and measured at the largest circular buffer size of 1,200 m (24).

### Data Analyses

Pearson's correlation coefficients assessed the correlation between NSES Index values at differing buffer sizes within each buffer shape. Next, correlations of NSES Index between buffer shapes (circular vs. network buffers) were computed at each buffer size, similar to other research (25). Bivariate analyses and multivariate analyses using survey weights examined the association of the NSES Index at different buffer sizes and shapes on the outcome measure of FV consumption to determine what spatial scale is of relevance for assessing the relationship of NSES and FV consumption. Multivariate analyses adjusted for age, gender, educational attainment, race/ethnicity, and urbanicity. Multivariate linear regression models were further stratified by gender. Since data were gathered from various geographic regions throughout the US, only 3.96% of census tracts in our study had more than one participant, thus, clustering within a census tract was negligible. Therefore, multilevel analyses were not included in this study. All analyses were conducted in STATA SE13 and weighted using survey weights (18). Analyses were evaluated against a criterion of α = 0.05.

## Results

Table 1 presents participants' demographic characteristics and home neighborhood characteristics for a circular 1,200 m buffer, which is the largest land area captured in the buffer measures. Examining NSES by quintiles shows that over 41% of the analytic sample lived in a medium/high NSES or high NSES neighborhood. On average, participants consumed FVs 2.2 times per day. Most participants were between the ages of 35–59. Approximately 70% of the sample was Non-Hispanic White, 17% Black, and 7% Hispanic. Over 35% of the sample had some college experience, and 46% had a 4-year college degree or higher. Most individuals resided in urban (42%) or suburban areas (41%), compared to rural areas (17%).

**Table 1:** Unweighted sample characteristics (*n* = 1,600)^*^ included in analysis from the National Cancer Institute's FLASHE Study.

**Characteristics**	**Percent (*n*)**
**Neighborhood socioeconomic status quintile (Circular 1,200 m distance from home)**	
Low	14.8% (236)
Medium/low	20.2% (323)
Medium	23.8% (380)
Medium/high	23.1% (370)
High	18.2% (291)
Fruit and vegetable consumption (daily frequency) (mean, standard error)	2.21 (0.041)
**Age**	
Ages 18–34	11.4% (182)
Ages 35–44	43.4% (694)
Ages 45–59	42.1% (674)
Ages 60+	3.1% (50)
**Gender**	
Male	26.8% (428)
Female	73.2% (1,172)
**Race/ethnicity**	
Hispanic	7.3% (116)
Non-Hispanic Black alone	17.3% (276)
Non-Hispanic White alone	69.7% (1,115)
Other	5.8% (93)
**Educational attainment**	
A high school degree, GED, or less	18.4% (295)
Some college but not a college degree	35.3% (565)
A 4-year college degree or higher	46.3% (740)
**Urbanicity (Circular 1,200 m distance from home)**	
Urban	42.3% (674)
Suburban	40.5% (651)
Rural	17.2% (275)

* *Sample is limited to those not missing for neighborhood socioeconomic status and key demographic factors of parent education, gender, race/ethnicity, and urbanicity. Sample source: Family Life, Activity, Sun, Health, and Eating Study (FLASHE) study*.

NSES values were highly correlated across all buffer sizes for both circular and street network buffers [Pearson correlations (*r*s) ≥ 0.98, *p*-values (*p*s) < 0.01; see Table 2]. Correlations between buffer measures and census tract level NSES were also very strong (*r*s ≥ 0.96, *p*s < 0.01). NSES values in circular neighborhood buffers also correlated with values for street network buffers of the same distance (*r*s ≥ 0.99, *p*s < 0.01), indicating that the NSES measure is similarly captured across circular and street buffers.

**Table 2:** Pearson's correlations of neighborhood socioeconomic status index values at various circular and street network buffer sizes, and census tract level; *n* = 1,600^*^ from the National Cancer Institute's FLASHE Study.

	**Circular buffer sizes**						**Street network buffer sizes**						**Census tract**
	**400 m**	**500 m**	**750 m**	**800 m**	**1,000 m**	**1,200 m**	**400 m**	**500 m**	**750 m**	**800 m**	**1,000 m**	**1,200 m**	
400 m	1						1						
500 m	0.999	1					1	1					
750 m	0.994	0.997	1				0.996	0.998	1				
800 m	0.993	0.996	1	1			0.995	0.997	1	1			
1,000 m	0.987	0.991	0.998	0.999	1		0.991	0.993	0.998	0.999	1		
1,200 m	0.981	0.985	0.994	0.996	0.999	1	0.986	0.988	0.995	0.996	0.999	1	
Census tract	0.986	0.983	0.974	0.972	0.965	0.957	0.99	0.988	0.983	0.982	0.977	0.971	1

* *All correlations significant at p < 0.01*.

Unadjusted model results (not shown) present a positive relationship between NSES and FV consumption frequency for all buffer sizes and shapes (β range: 0.12–0.15; *p*s < 0.05). The relationship of NSES and FV consumption frequency also remained significant when using census tracts as a measure of NSES (β = 0.13, *p* = 0.047). When stratified by gender, unadjusted models are only significant for women (βs = 0.19–0.25, *p*s ≤ 0.001).

In a multivariable model controlling for individual-level demographics (Table 3), NSES at various buffer sizes and shapes was not significantly associated with FV consumption frequency among the full sample (βs = 0.01–0.05, *p*s = 0.49–0.84). However, in stratified models, greater NSES was generally associated with greater frequency of FV consumption among women, using most NSES buffer distances and sizes. All NSES measurements using a circular buffer were significantly associated with FV consumption frequency in women (βs = 0.13–0.18, *p*s = 0.01–0.04). Street buffer measures of NSES were only significantly associated with women's FV consumption frequency at distances of 750 m or greater (βs = 0.13–0.16, *p*s = 0.02–0.04). No associations between NSES at the various buffer measures and FV consumption frequency were statistically significant among men (βs = −0.07 to −0.11, *p*s = 0.39–0.58). Census tract NSES was not associated with FV consumption in any of the multivariable models (βs = −0.10 to 0.11, *p*s = 0.07–0.84).

**Table 3:** Multivariable linear regressions predicting fruit/vegetable consumption frequency from neighborhood socioeconomic status index across buffer size, buffer shape, and gender^*^, *n* = 1,600 from the National Cancer Institute's FLASHE Study.

NSES at various buffers^*^	Full model^*^			**Male (*****n*** **=** 428)^*^			**Female (*****n*** **=** 1,172)^*^		
	**Beta (SE)**	**CI**	***p***	**Beta (SE)**	**CI**	***p***	**Beta (SE)**	**CI**	***p***
400 m circular buffer	0.02 (0.07)	(−0.10, 0.15)	0.71	−0.10 (0.12)	(−0.34, 0.15)	0.44	**0.13 (0.06)**	**(0.01, 0.25)**	**0.04**
400 m street buffer	0.02 (0.06)	(−0.11, 0.15)	0.76	−0.10 (0.12)	(−0.34, 0.13)	0.40	0.13 (0.06)	(0.01, 0.25)	0.05
500 m circular buffer	0.03 (0.07)	(−0.10, 0.16)	0.66	−0.09 (0.12)	(−0.34, 0.15)	0.45	**0.14 (0.06)**	**(0.01, 0.26)**	**0.03**
500 m street buffer	0.02 (0.06)	(−0.11, 0.15)	0.78	−0.10 (0.12)	(−0.34, 0.13)	0.39	0.12 (0.06)	(0.01, 0.25)	0.05
750 m circular buffer	0.05 (0.07)	(−0.09, 0.18)	0.50	−0.08 (0.13)	(−0.33, 0.17)	0.52	**0.16 (0.07)**	**(0.03, 0.29)**	**0.02**
750 m street buffer	0.02 (0.07)	(−0.11, 0.15)	0.75	−0.11 (0.12)	(−0.35, 0.13)	0.39	**0.13 (0.06)**	**(0.01, 0.26)**	**0.04**
800 m circular buffer	0.05 (0.07)	(−0.09, 0.18)	0.49	−0.08 (0.13)	(−0.33, 0.17)	0.51	**0.16 (0.07)**	**(0.03, 0.29)**	**0.01**
800 m street buffer	0.02 (0.07)	(−0.11, 0.15)	0.73	−0.11 (0.12)	(−0.34, 0.13)	0.39	**0.13 (0.06)**	**(0.01, 0.26)**	**0.04**
1,000 m circular buffer	0.05 (0.07)	(−0.08, 0.19)	0.43	−0.08 (0.13)	(−0.33, 0.18)	0.55	**0.17 (0.07)**	**(0.04, 0.30)**	**0.01**
1,000 m street buffer	0.03 (0.07)	(−0.1, 0.16)	0.63	−0.10 (0.12)	(−0.34, 0.14)	0.42	**0.15 (0.07)**	**(0.02, 0.27)**	**0.02**
1,200 m circular buffer	0.06 (0.07)	(−0.08, 0.20)	0.38	−0.07 (0.13)	(−0.33, 0.18)	0.58	**0.18 (0.07)**	**(0.04, 0.31)**	**0.01**
1,200 m street buffer	0.04 (0.07)	(−0.09, 0.17)	0.53	−0.09 (0.12)	(−0.33, 0.15)	0.47	**0.16 (0.07)**	**(0.03, 0.29)**	**0.02**
Census tract NSES	0.01 (0.06)	(−0.11, 0.14)	0.84	−0.10 (0.12)	(−0.34, 0.13)	0.39	0.11 (0.06)	(−0.01,0.23)	0.07

* *Models control for individual level age, race/ethnicity, gender, educational attainment and urbanicity; bolded values are significant at p < 0.05; dependent variable is estimated daily frequency of consuming fruits, salad, and other vegetables*.

*NSES, Neighborhood socioeconomic status*.

## Discussion

The publicly available GeoFLASHE dataset includes contextual variables that were computed for a range of neighborhood definitions based on distance from the participant's home that varied on buffer shape (street network and circular) and size (400–1,200 m), allowing analysts to select the appropriate option for their research questions (20). The present study aimed to examine the buffer size(s) and shape(s) that are important for understanding the association between neighborhood SES data and adult FV consumption. Factor scores for neighborhood socioeconomic status were highly correlated across the different buffer shapes and sizes defining home neighborhoods. This stability is likely due to bordering census tracts having similar socioeconomic profiles. Importantly, and an area for future research, this may or may not be the case for other specific contextual analyses. For example, built environment measures may be more likely to differ at a smaller geographic level.

In both unadjusted models and models adjusted for sociodemographics, NSES predicted frequency of FV consumption among a subsample of women in FLASHE. When controlling for demographics, this association was significant in almost all analyses, except when analyzed at the smallest (400–500 m) street network buffers. These associations were not significant among men or when neighborhood was calculated at the census tract level. Research trajectories in this area can focus on further study of mechanisms underlying associations between NSES and FV consumption. One possibility is that these associations are explained by greater consumer access to fruits and vegetables at higher levels of NSES. Prior research has found that individuals living in low NSES neighborhoods have less access to supermarkets and greater access to convenience stores, variables which have been associated with consuming fewer servings of FVs per day (3, 26). Effects among women specifically may be related to gender differences in food shopping and psychosocial variables (15, 16). Women are more likely than men to do food shopping (15), such that NSES (and related, food store accessibility) may be more impactful for women's purchases of the FVs that they will consume. Our findings align with previous research showing differences in the relationship of neighborhood level factors and related health domains by gender (13, 14). Psychosocial variables may also contribute to these findings and should be included in future studies, particularly in longitudinal research. For example, women's greater FV consumption may be partially explained by gender differences in psychosocial variables related to eating (such as attitudes and perceived behavioral control) (16).

Considered in conjunction with other research, the present study highlights that there may be important differences in findings across studies that use various methods of defining a neighborhood. Unlike our study which found that the NSES-FV consumption association did not hold for the full sample in adjusted models, a national study using NHANES data (which did not stratify by gender) found a positive association between these variables when NSES was defined at the census tract level (4). Another analysis of a large US sample did not show a strong association between NSES and FV consumption, when NSES was defined as median household income (27). Our results show that the association of NSES and FV consumption varies slightly by neighborhood definition for women, with stronger associations at larger neighborhood sizes. Additionally, census tracts using the administratively defined boundary were not as sensitive to the FV consumption outcome. The neighborhood definitions that best describe the role of NSES for FV consumption may be those that encompass a larger neighborhood size, if the larger neighborhood definitions more accurately reflect a person's exposure to neighborhood resources, such as supermarkets. Whether or not neighborhood definitions account for street connectivity (e.g., street network buffers as compared to circular buffers) may be less impactful for examining NSES—FV associations. Researchers should be mindful of the spatial area that is appropriate for their research, *a priori*. Even though neighborhood configurations did not substantially account for different findings in the current analysis, this is likely to vary by the specific neighborhood predictor(s) and behavioral outcome(s) of interest. NSES may (for example) be more relevant at a smaller scale for physical activity, given that issues of neighborhood safety and walkability are associated with physical activity (28). For researchers interested in specific subcomponents of NSES, it is possible that different indicators of the NSES composite score (e.g., median income or education) may vary at different neighborhood definitions. Also, neighborhood variables about access of resources or locations supporting healthy eating and physical activity that may be farther from the home may benefit from analysis of a larger spatial area that uses a street network definition for distance to reflect the route one would walk or drive to a location (20).

Other methodological considerations focus on innovative approaches for capturing one's experience with their neighborhood, such as using GPS coordinates that highlight activity spaces or actual geographic area traveled by the individual during the course of the day (29). Capturing activity spaces is more resource intensive than using buffers surrounding an individual's home address. However, given recent strides in technology with most individuals having access to smartphones, it may not be as onerous as in the past and has been used in assessing other health behaviors such as physical activity (30). Another method for capturing an individual's neighborhood environment may involve self-reported mapping of the individual's neighborhood, to capture what they perceive as their neighborhood boundary. This method may also be time and resource sensitive, limiting its use in large scale studies. However, recent literature in physical activity research indicates that residential buffers around an individual's home may not be the most relevant geographic boundary for capturing where individuals participate in physical activity, and thus, other methods of capturing the neighborhood may be important for understanding other health behaviors, including dietary behaviors (31).

The study of neighborhood definitions is also important for intervention development. Interventions may be easier to implement on a geographic boundary commonly used, such as administrative boundaries, rather than by buffer areas surrounding an individual's home. For example, interventions aimed at introducing farmer's markets or incentivizing fruit and vegetable purchasing at farmer's markets may be easier to implement in predefined neighborhoods than in geographic areas that have a greater percent of individuals with lower FV consumption (32). However, understanding relevant geospatial scale when assessing NSES Index and FV consumption, or associations between any neighborhood variable and health behaviors, may facilitate a cross-connect between scientific research identifying associations and the implementation of community-based or policy interventions, and inform what geographic scale to ideally intervene upon.

A limitation of this study is that it is cross-sectional, such that we cannot determine the causality of neighborhood influence on fruit and vegetable consumption. Participants in FLASHE also tended to report a relatively high socioeconomic status which may affect some of the associations examined in this paper. A second limitation is the issue of residential mobility. Individuals who have a higher individual-level educational attainment or income may self-select to live in higher SES neighborhoods, or in neighborhoods with large supermarkets and greater healthy food access, thus reducing variability in NSES for this study. We also were not able to account for other neighborhood or county level factors that may influence FV consumption. Future studies should also measure other neighborhood level factors, such as the retail food environment, or the actual locations where participants purchase foods. Research indicates that individuals may purchase food near their workplace (33), suggesting that NSES surrounding a workplace may be an important predictor of food purchasing that was not included in FLASHE. Another future research opportunity involves examining comprehensive models that include both the home neighborhood insights gained from this paper and additional variables of the individual (age groups, psychosocial factors, physical activity behaviors), dyad (family), and other environments (e.g., school, workplace) to further investigate moderators and mediators of the neighborhood-FV association for both adults and families. Another limitation of this study is that although we are aiming to examine differences in gender, a question on sex was used as a proxy for the gender construct. Future research should examine differences by sex and gender separately, and identify any nuances between the two measures in relation to fruit and vegetable intake.

Despite these limitations, this study contributes to our understanding of neighborhood level influences on FV consumption frequency. This study extends current research on neighborhood SES and allows for a sensitivity analyses of neighborhood size and shape. The use of several buffer sizes and shapes allows for a comparison of effect sizes across multiple different metrics of NSES. Additionally, this study accounted for the percentages of the buffer area on census tracts, allowing for a potentially more accurate account of the NSES around an individual's home, rather than just capturing the NSES of the closest census tract. This approach may be especially important in urban areas, where census tracts with varying census demographics can be adjacent to one another.

## Conclusion

The current study extends previous work in other fields, like physical activity research, and contributes to the understanding of buffer size and shape differences in relation to NSES and FV consumption. Findings suggest that NSES did not vary across buffer sizes or shapes in the publicly available GeoFLASHE dataset and that the role of neighborhood context for dietary consumption may operate differently by gender. Understanding the relevant size and shape of neighborhoods for health behaviors may improve our understanding of how neighborhood access to resources may influence those behaviors, such as FV consumption. Furthermore, understanding how neighborhoods are defined can better inform policies and interventions aimed at improving FV consumption.

## Data Availability Statement

Publicly available datasets were analyzed in this study. This data can be found here: https://cancercontrol.cancer.gov/brp/hbrb/flashe-study.

## Ethics Statement

The studies involving human participants were reviewed and approved by the Institutional Review Boards of Westat, Inc., and the National Cancer Institute. The patients/participants provided their written informed consent to participate in this study.

## Author Contributions

MP, AO, DS, MY, and LN: conceptualization. MP, DS, BL, and MY: analysis. MP, LD, HD'A, and AO: writing. MP, AO, LD, HD'A, DS, BL, MY, and LN: review/editing. All authors approved the submitted version.

## Funding

The Family Life, Activity, Sun, Health, and Eating (FLASHE) Study was funded by the National Cancer Institute under contract number HHSN261201200039I issued to Westat.

## Conflict of Interest

DS was employed by company Westat, Inc. The remaining authors declare that the research was conducted in the absence of any commercial or financial relationships that could be construed as a potential conflict of interest.

## Publisher's Note

All claims expressed in this article are solely those of the authors and do not necessarily represent those of their affiliated organizations, or those of the publisher, the editors and the reviewers. Any product that may be evaluated in this article, or claim that may be made by its manufacturer, is not guaranteed or endorsed by the publisher.
